# Analytical Property of Scattering Matrix:Spectroscopy Phenomena and Sharp Overlapping Autoionization Resonances

**DOI:** 10.1038/s41598-017-11823-8

**Published:** 2017-09-14

**Authors:** Rui Jin, Xiao-Ying Han, Xiang Gao, De-ling Zeng, Jia-Ming Li

**Affiliations:** 1 Department of Physics and Center for Atomic and Molecular Nanosciences, Tsinghua University, Beijing, 100084 China; 2 Beijing Computational Science Research Center, Beijing, 100193 China; 3 Institute of Applied Physics and Computational Mathematics, Beijing, 100088 China; 4 Key Laboratory for Laser Plasmas (Ministry of Education) and Department of Physics and Astronomy, Shanghai Jiao Tong University, Shanghai, 200240 China; 5 Collaborative Innovation Center of Quantum Matter, Beijing, 100084 China

## Abstract

An extended atomic data base with sufficiently high precision is required in astrophysics studies and the energy researches. For example, there are “infinite” energy levels in discrete energy region as well as overlapping resonances in autoionization region. We show in this paper the merits of our relativistic eigenchannel R-matrix method R-R-Eigen based on the analytical continuation properties of scattering matrices for the calculations of the energy levels, overlapping resonances and the related transitions. Using Ne^+^ as an illustration example, the scattering matrices of Ne^+^ in both discrete and continuum energy regions are calculated by our R-R-Eigen method directly. Based on our proposed projected high dimensional quantum-defect graph (symmetrized), one can readily determine the accuracies of the calculated scattering matrices using the experimental energy levels in a systematical way. The calculated resonant photoionization cross sections in the autoionization region are in excellent agreement with the benchmark high resolution experiments. With the scattering matrices checked/calibrated against spectroscopy data in both discrete and continuum energy regions, the relevant dynamical processes should be calculated with adequate accuracies. It should then satisfy the needs of the astrophysical and energy researches.

## Introduction

In astrophysical and fusion energy researches, the atomic data such as energy levels, collision cross sections, photoionization cross sections, dielectronic recombination rates and transition rates of atoms with sufficient precision are needed^1–8^. For instance, the optical recombination lines (ORLs) and collisional excitation lines (CELs) are both used to determine the abundances of metal elements (such as Oxygen) in Planetary nebular (PNe)^1–3^. While the metal abundances determined from the ORLs are much higher than that determined by CELs sometimes^1^, showing the strong dependence on the precision of related atomic data, especially the dielectronic recombination rates with appropriate cascading correction. It is generally accepted that the R-matrix type methods are good candidates to obtain the required atomic data^9–15^. But for the conventional R-matrix method: 1) it’s hard to assure the accuracies for each level/resonance^16, 17^; 2) a very fine energy grid is needed to guarantee all the levels/resonances without missing any lines; 3) it’s not trivial to determine and assign all the levels and resonances precisely.

Based on the analytical continuation properties of the short range scattering matrices, there exist intimate relations between atomic energy levels and the related electron-ion collision processes^18–33^. According to this property, we have proposed a scenario to provide such large scale atomic data with enough physical precisions which can be comparable with spectroscopic accuracies^18–23^. In our scenario, the short range scattering matrices (i.e., physical parameters in multi-channel quantum defect theory (MQDT)^24–33^ as well as corresponding wave functions in both bound and continuum energy regions can be calculated directly with high accuracy by our recent developed codes, i.e., R-Eigen code (Eigen-channel based on non-relativistic R-matrix method) and R-R-Eigen code (Eigen-channel based on relativistic R-matrix method)^18–23^. By applying the MQDT, one can calculate and predict all energy level positions up to fine-structures in discrete energy regions (i.e., bound state energy regions) without missing any lines “semi-analytically”. On the other hand, the short range scattering matrices in the discrete energy regions can be examined stringently by precise spectroscopic data experimentally. Therefore, accuracies of the MQDT parameters (i.e., scattering matrices) in bound energy regions can be readily ascertained. Through analytical properties of short range scattering matrices, the scattering matrices in continuum energy regions can be obtained with desired accuracies. One can then obtain relevant cross sections for electron-ion collision with similar accuracy. Note that the short range scattering matrices vary smoothly with energy because of their analytical continuation property. Therefore, one only need to calculate the short range scattering matrices in a few sample energy grids over the energy regions of interest, which is one merit of the R-R-Eigen method. The details will be given in the next section.

In present paper, we exhibit the merits of our R-R-Eigen method in the complex overlapping resonances for Ne^+^ photoionization processes (i.e., the inverse process of the dielectronic recombinations). Using all available precision spectroscopic data to calibrate our calculated scattering matrices and the corresponding dipole transition matrix elements in the discrete energy region, the resonant photoionization cross sections are calculated in the autoionization region. They are in excellent agreement with the benchmark high resolution experiments conducted at the synchrotron radiation light source, i.e., the Advanced Light Source (ALS) at Lawrence Berkeley National Laboratory. It should be noted that the conventional R-matrix method results reported therein^34^ are only in a fair agreement with the experiments. The origins of all the overlapping resonances in the experimental energy regions are assigned at the same time. Furthermore, in the calibration processes of the scattering matrices, we proposed a graphical method, i.e., projected high dimensional quantum-defect graph (symmetrized), to compare the theoretical energy levels with all the spectroscopic data in a systematical way readily for general multi-thresholds (more than two) cases. This is an extension of the Lu-Fano plot^35, 36^ valid only for two-thresholds cases and can be applied for any general atoms. With this method, one should be able to provide various accurate atomic data such as photoionization rates, dielectronic recombination rates for any atoms (ions). Hopefully, with all necessary atomic data calculated with the method, the R-R-Eigen method should be indispensable in the study of basic dynamic processes in astrophysics and laboratory plasmas.

## Results and Discussions

### Relativistic eigenchannel R-matrix method (R-R-Eigen method)

The N + 1 electron system consisting of an N-electron target atom and an excited electron can be calculated using the R-matrix type method^9–15^, which has been successfully developed as an ab initio method for treating a variety of dynamic processes in atomic physics. Let’s briefly review the relativistic eigenchannel R-matrix method (i.e., the R-R-Eigen code)^18^, which mainly differs from the traditional R-matrix method^9–15^ by the definitions of physical (ionization) channels, including the opened channels and some relevant closed channels(i.e. *n*_*p*_). Other closed channels for much higher thresholds with deep negative orbital energies and the *N* + 1 bound type configurations are defined as closed computational channels(i.e. *n*_*com*_), which are included in our calculations to assure electron correlations taken into account adequately. More specifically, with *n*_*p*_ physical (ionization) channels and *n*_*com*_ computational channels for symmetry block with total angular momentum *J* and parity *π*, the *n*_*p*_ × *n*_*p*_ short-range reaction matrices ${\bar{K}}^{{J}^{\pi }}(E)$ (equivalent to scattering matrices by ${\bar{S}}^{{J}^{\pi }}=(1+i{\bar{K}}^{{J}^{\pi }}){(1-i{\bar{K}}^{{J}^{\pi }})}^{-1}$)^37^ are calculated at *r*_0_ = *r*_*N*+1_ as,

1 $${{\rm{\Psi }}}_{i}^{{J}^{\pi }}(E,{r}_{{\rm{0}}})={{\rm{\Phi }}}_{i}\,{f}_{i}({r}_{{\rm{0}}},E)-\sum _{j={\rm{1}}}^{{n}_{p}}{{\rm{\Phi }}}_{j}{g}_{j}({r}_{{\rm{0}}},E){\overline{K}}_{ij}^{{J}^{\pi }}+\sum _{j={n}_{p}+1}^{{n}_{p}+{n}_{com}}{{\rm{\Phi }}}_{j}{{\rm{\Theta }}}_{j}({r}_{{\rm{0}}},E),\,i\le {n}_{p}.$$

For the *i* ^*th*^ physical (ionization) channel associated with the target state in Φ_*i*_, the relativistic regular and irregular Coulomb wave functions *f*_*i*_(*r*, *E*), and *g*_*i*_(*r*, *E*), cover the entire energy ranges of one-electron orbitals, i.e., *ε*_*l*_ = *E* − *I*_*i*_ > −*q* ^2^/*l* ^2^ (except for *l* = 0, ${\varepsilon }_{l=0} > {\varepsilon }_{l=0}^{scf}$, in Ryd.). Here *I*_*i*_ is the threshold of the ion core in Φ_*i*_, *l* is the angular momentum of the excited channel electron and *q* is the charge of a long-range potential^30, 33^. Because of these energy criteria, the number of physical channels will increase as energy increases, with total number of channels *n*_*tot*_ = *n*_*com*_ + *n*_*p*_ unchanged in a specific calculation with considered targets. For both the discrete and continuous energy regions of interest, the physical parameters for MQDT, i.e., *n*_*p*_ eigen-quantum-defects *μ*_*α*_(*α* as eigenchannel index) and a *n*_*p*_ × *n*_*p*_ orthogonal transformation matrix *U*_*iα*_, can then be calculated by diagonalizing the short range scattering matrices,

2 $${\overline{S}}_{ij}^{{J}^{\pi }}=\sum _{\alpha }{U}_{i\alpha }\exp (i2\pi {\mu }_{\alpha }){U}_{j\alpha },$$

where the *i*, *j* denote the physical (ionization) channel indexes and the *U*_*iα*_ can be represented by ${n}_{p}({n}_{p}-1)/2$ Euler-type angles *θ*_*k*_^27^.

### The calculation of scattering matrices for Ne^+^*J*^*π*^ = 1/2^+^ and the analytical properties

**Figure 1:**
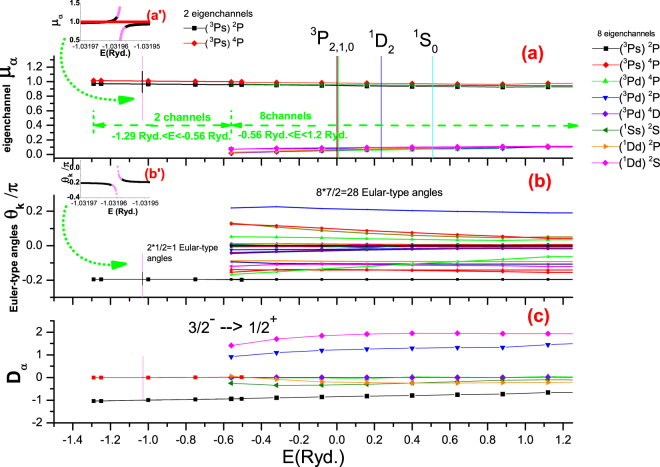
Eigenchannel calculations for ${J}^{\pi }={1/2}^{+}$ final channel of Ne^+^ in the two-channel energy region (−1.29 ≤ *E* ≤ −0.56*Ryd*.) with the two physical (ionization) channels $[{}^{3}P_{1}{s}_{1/2},{}^{3}P_{0}{s}_{1/2}]$ and in the eight-channel energy region (−0.56 ≤ *E* ≤ 1.2*Ryd*.) with the eight physical (ionization) channels [${}^{3}P_{1}{s}_{1/2}$, ${}^{3}P_{0}{s}_{1/2}$, ${}^{3}P_{2}{d}_{3/2}$, ${}^{3}P_{1}{d}_{3/2}$, ${}^{3}P_{2}{d}_{5/2}$, ${}^{1}D_{2}{d}_{3/2}$, ${}^{1}D_{2}{d}_{5/2}$, ${}^{1}S_{0}{s}_{1/2}$], respectively. (**a**) The eigenchannel quantum defects *μ*_*α*_ of the two eigen-channels and of the eight eigen-channels denoted in the legend respectively. The values were calculated by the R-R-Eigen code and calibrated with precise spectroscopy data. Note an isolated sharp resonance state $2{s}^{1}2{p}^{6}({}^{2}S_{1/2})$ at *E* = −1.03*Ryd*. is denoted as a vertical light-magenta curve. Smooth variations and connections between the two eigenchannels and the eight eigenchannels demonstrate the analytical continuation properties of the scattering matrices. (**b**) The Euler-type angles representing the transformation matrices *U*_*iα*_. (**c**) The eigenchannel dipole-matrix element *D*_*α*_ from the initial state ${}^{2}P_{3/2}^{o}$.

In the Ne^+^($2{s}^{2}2{p}^{5}\,{}^{2}P_{3/2,1/2}^{o}$) photoionization processes, the possible final channel symmetries can be ${J}^{\pi }=1/{2}^{+},3/{2}^{+},5/{2}^{+}$. As an example, for the ${J}^{\pi }=1/{2}^{+}$, there are five thresholds i.e. Ne^2+^ ($2{s}^{2}2{p}^{4}\,{}^{3}P_{2,1,0}^{e},{}^{1}D_{2}^{e}\,{\rm{and}}{}^{1}{S}_{0}^{e}$) for five target states and some much higher thresholds [(i.e. Ne^2+^ ($2{s}^{1}2{p}^{5}{}^{3}{P}_{2,1,0}^{o}\,{\rm{and}}{}^{1}{P}_{1}^{o}$)] associated with seven computational channels in present R-R-Eigen calculations (total channel *n*_*tot*_ = 15). The number of physical (ionization) channels *n*_*p*_ will change with energy as shown in Fig. 1, i.e., in the two-channel region, there are the two eigen-quantum-defects *μ*_*α*_ and the 2(2–1)/2 = 1 Euler-type angle for the two eigenchannels $[({}^{3}Ps){}^{2}P,({}^{3}Ps){}^{4}P]$; in the eight-channel region, there are the eight eigen-quantum defects *μ*_*α*_ and the ${n}_{p}({n}_{p}-1)/2=28$ Euler-type angles for the eight eigenchannels [$({}^{3}Ps){}^{2}P,({}^{3}Ps){}^{4}P$, $({}^{3}Pd){}^{4}P,({}^{3}Pd){}^{2}P,({}^{3}Pd){}^{4}D,$ $({}^{1}Ss){}^{2}S$, $({}^{1}Dd){}^{2}P$, $({}^{1}Dd){}^{2}S$] shown in Fig. 1(a) and (b) respectively. Furthermore, the calculated eigenchannel parameters in 2-channel energy region smoothly connect with those in 8-channel energy region^23^. Therefore one only needs to calculate the short range reaction matrices (i.e., short range scattering matrices) at a few energy grids for entire energy regions. The corresponding eigenchannel wavefunctions Ψ_*α*_ normalized per unit energy can then be calculated directly. Hence, the corresponding dipole transition matrix elements *D*_*α*_ for photoionization processes from the initial states (such as ${}^{2}P_{3/2}^{o}$) can also be obtained as shown in Fig. 1(c). Note that a sharp resonance due to the isolated state $2{s}^{1}2{p}^{6}({}^{2}S_{1/2})$ is denoted as a vertical light-magenta curve at *E* = −1.03*Ryd*., whose position is determined by judiciously adjusting the relevant *μ*_*α*_ against the experiment value (the details will be reported elsewhere). Note that one can improve the calculation accuracies either by increasing the computational channels to include more electron correlations or calibrating the scattering matrices with the precise spectroscopy experimental data. The calibrated *μ*_*α*_ are shown in Fig. 1(a), which will be discussed later.

### Calibration of the scattering matrices with precise spectroscopic data

In order to demonstrate how to calibrate our results of R-R-Eigen calculations with the available precise spectroscopic data, we start with the eigenchannel wave-functions Ψ_*α*_ outside the reaction zone (i.e., *r* ≥ *r*_0_)^18, 25–33^,

3a $${{\rm{\Psi }}}_{\alpha }^{{J}^{\pi }}=\sum _{i=1}^{{N}_{p}}{{\rm{\Phi }}}_{i}\cdot {U}_{i\alpha }\cdot ({f}_{i}\,\cos \,\pi {\mu }_{\alpha }-{g}_{i}\,\sin \,\pi {\mu }_{\alpha })\quad \,r\ge {r}_{0}$$

In the discrete energy region for the spectroscopic energy levels, the energy eigen wave-function (also for auto-ionization states) can be expressed as a superposition of eigenchannel wave-functions:

3b $${{\rm{\Psi }}}^{{J}^{\pi }}(E)=\sum _{\alpha }^{{N}_{p}}{A}_{\alpha }{{\rm{\Psi }}}_{\alpha }^{{J}^{\pi }}(E),$$

where *A*_*α*_ are determined by the asymptotic boundary conditions^18, 25–33^. The bound state asymptotic boundary conditions (i.e., *N*_*c*_ = *n*_*p*_, where all channels are closed) lead to,

4a $$\sum _{\alpha }^{{N}_{p}}{U}_{i\alpha }\,\sin \,\pi ({\nu }_{i}+{\mu }_{\alpha }){A}_{\alpha }^{\rho }=0\quad i\in {N}_{c}\,{\rm{closed}}\,{\rm{channels}},$$

with the *N*_*th*_ effective principal quantum numbers *ν*_*i*_ corresponding to *N*_*th*_ number of different thresholds *I*_*i*_ and spanning a *N*_*th*_-dimensional space, defined as,

4b $$E={I}_{i}-{q}^{2}/{\nu }_{i}^{2}\quad {\rm{in}}\,Ryd{\rm{.}},$$

with *q* = 2 for the Ne^+^ system. The existence of nontrivial *A*_*α*_ requires the vanishing of determinant of coefficient matrix:

5 $$F(\{{\nu }_{i}\},\{{U}_{i\alpha },{\mu }_{\alpha }\})={\rm{\det }}({U}_{i\alpha }\,\sin \,\pi ({\nu }_{i}+{\mu }_{\alpha }))={\rm{\det }}({F}_{i\alpha })=0.\,$$

To illustrate various features in the calibration process clearly, we’ll discuss two methods of the solution of Eqs (4b) and (5) in the following part of the section, i.e., 1) recursive projection method and 2) projected high dimensional quantum-defect graph (symmetrized).

#### Recursive projection method

From the geometric view, Eqs (4b) and (5) can be represented as a one-dimension curve and a (N_th_ − 1)-dimension surface $\{{\nu }_{i}(i=1,...,{N}_{th})\}$ in a N_th_ dimensional space respectively. For cases with ${N}_{th} > 2$, which are somewhat different from the case associated with only two thresholds^19–21, 25–28, 35, 36^, where the solutions of the Eqs (4b) and (5) can be represented graphically in a plot with the only two effective principal quantum numbers. More specifically, with only two thresholds, there are two sets of effective principal quantum numbers, *n*_1_ number of *ν*_1_ corresponding to the first threshold *I*_*one*_ and *n*_2_ number of *ν*_2_ corresponding to the second threshold *I*_*two*_ with *I*_*one*_ < *I*_*two*_. In the (*ν*_1_, *ν*_2_) plot, it is so called the Lu-Fano plot^35, 36^, where the *ν*_2_ can be regarded as known variables to scan the energy according to Eq. (4b) and the *ν*_1_ can be determined by solving the Eq. (5). In such a plot, there will be avoid-crossing curves basically consisting of *n*_1_ number of “horizontal” curves with *n*_2_ number of “vertical” resonances. The final solutions are then the crossing points of the curves and the one-dimensional energy curve according to Eq. (4b). With more than two thresholds, in order to semi-analytically and graphically calculate the discrete levels and compare with the experimental spectroscopy levels, the (*N*_*th*_ − 1)-dimensional surface should be projected onto a two-dimensional plots. According to physical requirements, we can select a pair of any two adjacent thresholds *I*_*one*_ and *I*_*two*_ with *I*_*one*_ < *I*_*two*_. Therefore there are two sets of effective principal quantum numbers: *n*_1_ number of *ν*_*i*_ forming a vector ${\overrightarrow{\nu }}_{1}$ ≡ (*ν*_*i*_; *I*_*i*_ ≤ *I*_*one*_) and *n*_2_ number of *ν*_*j*_ forming a vector ${\overrightarrow{\nu }}_{2}\equiv ({\nu }_{j};{I}_{j}\ge {I}_{two})$. To solve these equations at a specific energy E, the ${\overrightarrow{\nu }}_{2}$ are regarded as known variables based on Eq. (4b). Therefore any element of ${\overrightarrow{\nu }}_{2}$ can be used to represent the energy. The remaining ${\overrightarrow{\nu }}_{1}$ are unknown variables which should be solved from Eq. (5). In this procedure, we set all elements of ${\overrightarrow{\nu }}_{1}$ equal to a same unknown variable −*τ* and *n*_1_ number of solutions can be readily obtained numerically. The corresponding ${A}_{\alpha }^{\rho }$ for each solution *ρ*(*ρ* = 1, …, *n*_1_) are also solved. In this way, we can obtain a two-dimensional plot $({\nu }_{2}^{a}\,{\rm{vs}}\,\tau )$, where ${\nu }_{2}^{a}$ can be any element of ${\overrightarrow{\nu }}_{2}$ to represent the energy. It’s interesting to note that the solved *n*_1_ number of ${\tau }^{\rho }$ can be regarded as effective eigen-quantum-defects for effective physical channels connecting with collisional eigen phase shifts as the energy analytically extends into the autoionization continuum. The corresponding *n*_1_ × *n*_1_ effective transformation matrices^18, 27, 28, 33^ ${T}_{i^{\prime} \rho }$ are constructed from ${A}_{\alpha }^{\rho }$ by ${T}_{i^{\prime} \rho }={\sum }_{\alpha }^{{n}_{p}}{U}_{i^{\prime} \alpha }\,\cos \,\pi (-{\tau }_{\rho }+{\mu }_{\alpha }){A}_{\alpha }^{\rho }/{N}_{\rho }$, with the normalization factor ${N}_{\rho }=\sqrt{{\sum }_{i^{\prime} }^{{n}_{1}}{[{\sum }_{\alpha }{U}_{i^{\prime} \alpha }\cos \pi (-{\tau }_{\rho }+{\mu }_{\alpha }){A}_{\alpha }^{\rho }]}^{2}}$. Note that the physical MQDT parameters {*τ* ^*ρ*^ and *T*_*i*_′_*ρ*_} for effective physical channel (i.e, scattering matrices of effective physical channel) can also be calculated with the R-R-eigen code if one would choose the set of *n*_1_ (*n*_1_ < *n*_*p*_) number of physical channels (with the thresholds *I*_*i*_ ≤ *I*_*one*_). In contrast with the eigenchannel parameters of full physical channels, these effective eigenchannel parameters are not smooth functions with energy, which have various resonance structures related with ${\overrightarrow{\nu }}_{2}$. Therefore one should choose an appropriate set of physical channels to guarantee the analytical continuation property of scattering matrices, as shown in Fig. 1.

**Figure 2:**
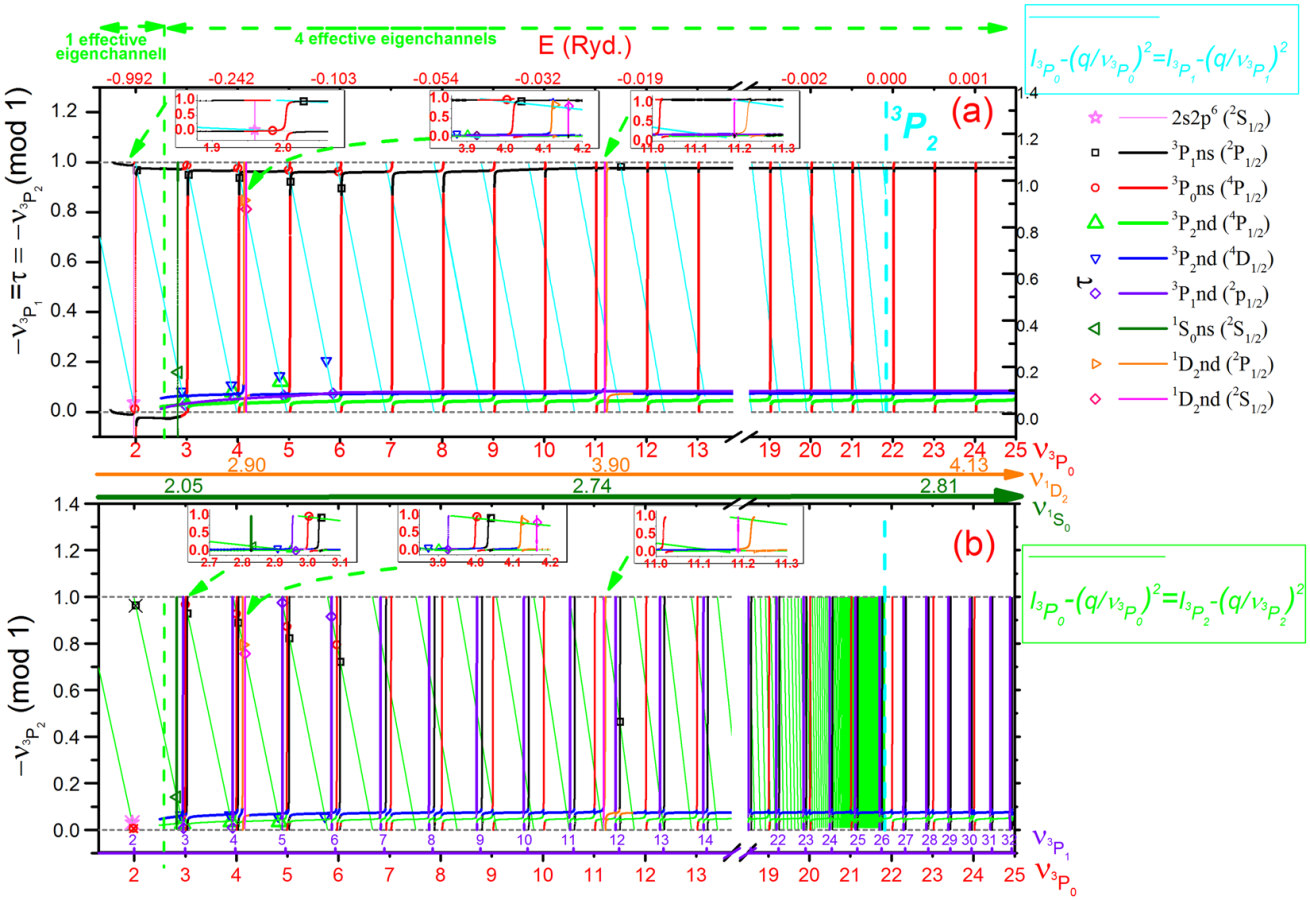
Graphical representation to solve Eq. (4b) and Eq. (5). (**a**) A projected two-dimensional graph $({\nu }_{{}^{3}P_{1}}\,{\rm{vs}}\,{\nu }_{{}^{3}P_{0}})$. Effective eigen-quantum-defects are shown as one (black) and four (black, blue, violet, green) branches of colored curves with sharp resonances for two-channel and eight-channel regions respectively. The auxiliary abscissas ${\nu }_{{}^{1}D_{2}}$ and ${\nu }_{{}^{1}S_{0}}$ are plotted so that one can easily see the isolated states in Fig. 2. Because of modulo 1, an equivalent auxiliary effective eigen-quantum-defect (i.e., $-{\nu }_{{}^{3}P_{{\rm{1}}}}=\tau  \sim 0$) is also plotted in the two-channel energy region from ${\nu }_{{}^{3}P_{0}}=1.5$ to ${\nu }_{{}^{3}P_{0}}=3$ to show its continuation into the four effective-eigenchannel region. Note that there is a sharp resonance at ${\nu }_{{}^{3}P_{0}}=2$ marked as light-magenta curve. (**b**) A recursive projected two-dimensional graph $({\nu }_{{}^{3}P_{2}}\,{\rm{vs}}\,{\nu }_{{}^{3}P_{1}})$ based on the effective eigen- quantum-defects as shown in (**a**) and the corresponding transformation matrices *T*_*i*_^′^_*ρ*_^18, 27, 28, 33^, by scanning the energies with the known ${{\nu }_{3}}_{{P}_{1}}$, and to solve the unknown variable ${\nu }_{{}^{3}P_{2}}$ in the Eq. (6) (i.e. the 1 × 1 determinant equations and the 4 × 4 determinant equations in the effective one-channel and the effective four-channel energy regions respectively). The abscissas ${{\nu }_{3}}_{{P}_{1}}$, ${{\nu }_{3}}_{{P}_{0}}$, ${{\nu }_{1}}_{{D}_{2}}$ and ${{\nu }_{1}}_{{S}_{0}}$ are all given in the figure for the convenience of charactering the relevant locally isolated resonances. The solution are three discrete levels marked as colored crosses for one-channel region and two (green and blue) branches of colored curves with sharp resonances for one-channel and four-channel regions respectively. The green line sections are energy relation according to Eq. (4b). The intersection points are the solutions of the Eqs (4b) and (6), corresponding to the observed experimental data^38^ denoted as colored symbols. Although all the observed Rydberg levels should be at the intersection points, it is not convenient to compare the precise spectroscopic data with the theoretical predications.

Let’s return to the Ne^+^ (${J}^{\pi }=1/{2}^{+}$) case as an illustration example. There are five effective principal quantum numbers (${\nu }_{{}^{3}{P}_{2}}$, ${{\nu }_{3}}_{{P}_{1}}$, ${{\nu }_{3}}_{{P}_{0}}$, ${{\nu }_{1}}_{{D}_{2}}$, ${{\nu }_{1}}_{{S}_{0}}$) associated with five thresholds of Ne^2+^ (2*p* ^4 3^ *P*_2,1,0_, ^1^ *D*_2_ and ^1^ *S*_0_). Because of the first two appearing strongly perturbed Rydberg series, ${}^{3}P_{0}ns({}^{4}P_{1/2})$ and ${}^{3}P_{1}ns({}^{2}P_{1/2})$, we first select ${I}_{one}={I}_{{}^{3}P_{1}}$, ${I}_{two}={I}_{{}^{3}P_{0}}$ and ${\nu }_{2}^{a}={\nu }_{{}^{3}P_{0}}$. With the eigenchannel MQDT parameters {*μ*_*α*_, *U*_*iα*_} in Fig. 1, the Eq. (5) is solved as the 2 × 2 and 8 × 8 determinant equations in the two-channel and the eight-channel energy regions respectively. For the eight-channel region (*n*_1_ = *n*_2_ = 4), the two dimensional plot (${\nu }_{{}^{3}P_{1}}\,{\rm{vs}}\,{\nu }_{{}^{3}P_{0}}$) is obtained by scanning energies with three known variables ${{\nu }_{3}}_{{P}_{0}}$, ${{\nu }_{1}}_{{D}_{2}}$ and ${{\nu }_{1}}_{{S}_{0}}$ according to Eq. (4b) and solving unknown variables $\tau =-{{\nu }_{3}}_{{P}_{1}}=-{{\nu }_{3}}_{{P}_{2}}$ from Eq. (5). Therefore, the solutions are basically four horizontal curves (black, blue, violet and green) with four types of vertical curves as resonances as shown in Fig. 2(a), which correspond to the four effective eigen-quantum-defects (*τ*_*ρ*_; *ρ* = 1, 2, 3, 4). The various colors of these curves shown in the figure represent the eigenchannel characters derived from the calculated ${A}_{\alpha }^{\rho }$ coefficients. Because we set ${\nu }_{2}^{a}={\nu }_{{}^{3}P_{0}}$ to represent energy, the ${}^{3}P_{0}ns({}^{4}P_{1/2})$ series form resonances at ${\nu }_{{}^{3}P_{0}}\approx {\rm{integer}}$, while the series associated with higher thresholds, i.e., ^1^ *D*_2_ and ${}^{1}S_{0}$ form locally isolated resonances, i.e., two pairs of orange and magenta curves $[({}^{1}D_{2}){\rm{3}},{\rm{4}}{d}_{3/2,5/2}\,\,{}^{2}P_{1/2},{}^{2}S_{1/2}]$ at ${\nu }_{{}^{1}D_{2}}\approx 2.9\,(i{\rm{.e}}{\rm{.}},{\nu }_{{}^{3}P_{0}}\approx 4)$ and ${\nu }_{{}^{1}D_{2}}\approx 3.9\,({\rm{i}}{\rm{.e}}.,\,{\nu }_{{}^{3}P_{0}}\approx \mathrm{11})$, and one dark-green vertical curve $({}^{1}S_{0}){\rm{3}}s{}^{2}{S}_{1/2}$ at ${\nu }_{{}^{1}S_{0}}\approx 2\,({\rm{i}}{\rm{.e}}.,\,{\nu }_{{}^{3}P_{0}}\approx 3)$. As the energy decreases to the two-channel region (*n*_1_ = *n*_2_ = 1), the two dimensional plot (${\nu }_{{}^{3}P_{1}}\,{\rm{vs}}\,{\nu }_{{}^{3}P_{0}}$) is obtained by scanning energies with one known ${\nu }_{{}^{3}P_{0}}$ according to Eq. (4b) and one unknown ${\nu }_{{}^{3}P_{1}}$ is solved from Eq. (5). In this case, only one resonant horizontal black curve corresponding to one effective-eigenchannel (*τ*_*ρ*_; *ρ* = 1) is solved, i.e., $-{\nu }_{{}^{3}P_{{\rm{1}}}}=\tau  \sim 1$. Because of modulo 1, an equivalent auxiliary effective eigen-quantum-defect ($-{\nu }_{{}^{3}P_{{\rm{1}}}}=\tau  \sim 0$) is also plotted in the two-channel energy region (i.e., $1.5\le {\nu }_{{}^{3}P_{0}}\le 3$) to show its continuation into the four effective-eigenchannel region. Note that an isolated sharp resonance marked as light-magenta vertical curve exists at ${\nu }_{{}^{3}P_{0}}\approx 1.96$, which originates from the $(2{s}^{1}2{p}^{6}){}^{2}S_{1/2}$ resonance in the eigen-quantum-defect as shown in Fig. 1. The observed Rydberg levels ${}^{3}P_{0}ns({}^{4}P_{1/2})$, ${}^{3}P_{1}ns({}^{2}P_{1/2})$ and ${}^{3}P_{1}nd({}^{2}P_{1/2})$ associated with the ${}^{3}P_{1}$ and ${}^{3}P_{0}$ thresholds are denoted as red open circles, black open squares and violet open diamonds. They are at the intersecting points between the effective eigen-quantum-defect curves and the cyan line sections representing the energy relation of ${I}_{{}^{3}P_{1}}-{q}^{2}/{\nu }_{{}^{3}P_{1}}^{2}={I}_{{}^{3}P_{0}}-{q}^{2}/{\nu }_{{}^{3}P_{0}}^{2}$. However, the other Rydberg levels, especially ${}^{3}P_{2}nd({}^{4}D_{1/2})$ and ${}^{3}P_{2}nd({}^{4}P_{1/2})$ associated with the ${}^{3}P_{2}$ threshold denoted as the blue and green open triangles respectively in Fig. 2(a), are not at the intersection points in this specific two-dimensional plots projected from the (*N*_*th*_ − 1)-dimensional surface.

From the point of view of effective eigenchannels, the wavefuncitons of discrete energy levels below the first threshold can also be expressed as the superposition of the effective eigenchannel wavefunctions. From the asymptotic boundary conditions of these discrete energy level wavefunctions, an equation similar to Eq. (5) is derived, which can be solved to obtain all energy levels below the first threshold. More specifically, in the present four effective physical channel region, there are four effective eigen-quantum-defects ${\tau }_{\rho }$ shown in Fig. 2(a) and the corresponding 4 × 4 effective transformation matrices ${T}_{i^{\prime} \rho }$ ^18, 27, 28, 33^. The boundary conditions now lead to,

6 $$F(\{{\nu }_{i^{\prime} }\},\{{T}_{i^{\prime} ,\rho },{\tau }_{\rho }\})={\rm{\det }}({T}_{i^{\prime} ,\rho }\,\sin \,\pi ({\nu }_{i^{\prime} }+{\tau }_{\rho }))={\rm{\det }}({F}_{i^{\prime} \rho })=0.$$

Because the effective four physical channels are just associated with ${}^{3}P_{1}$ and ${}^{3}P_{2}$, we select ${I}_{one}={I}_{{}^{3}P_{2}}$, ${I}_{two}={I}_{{}^{3}P_{1}}$ and ${\nu }_{2}^{a}={\nu }_{{}^{3}P_{1}}$ here. Then ${\overrightarrow{\nu }}_{1}$ and ${\overrightarrow{\nu }}_{2}$ vectors will simply become two scalars of ${\nu }_{{}^{3}P_{2}}$ and ${\nu }_{{}^{3}P_{1}}$ respectively. The Eq. (6) can be solved by scanning the energies with the known ${{\nu }_{3}}_{{P}_{1}}$, and set the only unknown variable $\tau =-{{\nu }_{3}}_{{P}_{2}}$. In this case, *n*_1_ = *n*_2_ = 2, the solutions are basically two horizontal curves (black and green) with sharp resonances, as shown in Fig. 2(b) [i.e., the (${\nu }_{{}^{3}P_{2}}\,{\rm{vs}}\,{\nu }_{{}^{3}P_{1}}$) plot]. Note that, since the abscissa of the plot is energy, we can choose other *ν*_*i*_ according to Eq. (4b) to represent energy. In order to elucidate the features of the plot clearly, both the ${{\nu }_{3}}_{{P}_{1}}$ and the ${\nu }_{{}^{3}P_{0}}$ in consistent with the one in Fig. 2(a) based on the energy relation ${I}_{{}^{3}P_{1}}-{q}^{2}/{\nu }_{{}^{3}P_{\,1}}^{2}={I}_{{}^{3}P_{0}}-{q}^{2}/{\nu }_{{}^{3}P_{0}}^{2}$, are plotted as the abscissas of Fig. 2(b). Compared with Fig. 2(a), there are six types of resonances. Two newly appeared series of ${}^{3}P_{1}ns({}^{2}P_{1/2})$ and ${}^{3}P_{1}nd\,({}^{2}P_{1/2})$ are associated with ${}^{3}P_{1}$ threshold, while the other four [i.e., ${}^{3}P_{0}ns({}^{4}P_{1/2})$, $({}^{1}D_{2})\,nd\,{(}^{2}{P}_{1/2}),({}^{1}D_{2})\,nd\,{(}^{2}{S}_{1/2})$ and $({}^{1}S_{0})\,ns\,{(}^{2}{S}_{1/2})$] are inherited from the effective eigen-quantum-defects shown in Fig. 2(a). Note that the positions of the resonances associated with ${}^{3}P_{1}$ are just near the integers of the abscissa ${\nu }_{{}^{3}{P}_{1}}$ shown in Fig. 2(b). For the one effective-eigenchannel region, it’s an effective single channel problem. The Eq. (6) becomes $\sin \,\pi ({\nu }_{{}^{3}P_{2}}+{\tau }_{\rho })=0$, and the solutions of Eq. (6) are three discrete solutions marked as crosses in Fig. 2(b), which are in excellent agreement with the first three experimental levels^38^.

In summary, these resonances belong to one isolated resonance and the six series of resonances with the corresponding six eigenchannel characters as shown in Fig. 2(b), i.e., one isolated resonance $[(2{s}^{1}2{p}^{6})\,{}^{2}S_{1/2};{\nu }_{{}^{3}P_{0}}\approx 1.96]$ marked as a light-magenta cross and the six series of resonances such as one periodic red vertical curves $[({}^{3}P_{0})ns{}^{4}P_{1/2};\,{\nu }_{{}^{3}P_{0}}\approx 2,\ldots ,25]$, one locally isolated resonances dark-green vertical curve $[({}^{1}S_{0})3s\,{}^{2}S_{1/2};\,{\nu }_{{}^{1}S_{0}}\approx 2]$, two pairs of orange and magenta curves $[({}^{1}D_{2})3,4{d}_{3/2,5/2}\,{}^{2}P_{1/2},{}^{2}S_{1/2};\,{\nu }_{{}^{1}D_{2}}\approx 2.9,3.9]$, and two quasi-periodic curves ($[({}^{3}P_{1})ns\,{}^{2}P_{1/2};\,{\nu }_{{}^{3}P_{1}}\approx 3,\ldots ,32]$ as black vertical curves and $[({}^{3}P_{1})\,nd\,{}^{2}P_{1/2};\,{\nu }_{{}^{3}P_{1}}\approx 3,\ldots ,32]$ as violet vertical curves). Note that the auxiliary abscissas ${{\nu }_{1}}_{{D}_{2}}$ and ${{\nu }_{1}}_{{S}_{0}}$ are given in Fig. 2 for the convenience to recognize the relevant resonances. As shown in Fig. 2(b), all the crossing points between the branch curves and the green line sections of [${I}_{{}^{3}P_{2}}-{q}^{2}/{\nu }_{{}^{3}P_{\,2}}^{2}={I}_{{}^{3}P_{0}}-{q}^{2}/{\nu }_{{}^{3}P_{0}}^{2}$] match the experimental observed levels^38^ marked as colored symbols. Therefore, we can compare with the observed precise spectroscopic data to check/calibrate the calculated scattering matrices in principle. However, it’s tedious and computational consuming to trace the branch curves by solving the MQDT equations, i.e., Eq. (6) with the four effective eigen-quantum-defects in Fig. 2(a). Furthermore, it is not convenient to compare with the observed spectroscopic data with the calculated crossing points clearly at the many sharp vertical resonance lines in Fig. 2(b).

#### Projected high dimensional quantum-defect graph (symmetrized)

Therefore, in order to obtain all the energy levels more clearly and conveniently, we propose a new projection method with additional constrain conditions. For the present Ne^+^ (${J}^{\pi }={1/2}^{+}$) case with ${I}_{one}={I}_{{}^{3}P_{1}}$, ${I}_{two}={I}_{{}^{3}P_{0}}$ and ${\nu }_{2}^{a}={\nu }_{{}^{3}P_{0}}$, the corresponding unknown variable vector is ${\overrightarrow{\nu }}_{1}=({\nu }_{{}^{3}P_{2}},{\nu }_{{}^{3}P_{1}})$ and known variables vector is ${\overrightarrow{\nu }}_{2}=({\nu }_{{}^{3}P_{0}},{\nu }_{{}^{1}D_{2}},{\nu }_{{}^{1}S_{0}})$. Note that in this new projection method, one can directly solve the related equations based on the eigenchannel parameters of the full physical channels (i.e., those smooth functions shown in Fig. 1), compared with the resonant parameters of the effective physical channels in the recursive projection method. We can choose additional constrain conditions between the two unknown variables ${\nu }_{{}^{3}P_{2}}$ and ${\nu }_{{}^{3}P_{1}}$ as,

7 $${\rm{\Delta }}\equiv {\nu }_{{}^{3}P_{2}}-{\nu }_{{}^{3}P_{1}}={[1/{\nu }_{{}^{3}P_{0}}^{2}-({I}_{{}^{3}P_{0}}-{I}_{{}^{3}P_{2}})/{q}^{2}]}^{-1/2}-{[1/{\nu }_{{}^{3}P_{0}}^{2}-({I}_{{}^{3}P_{0}}-{I}_{{}^{3}P_{1}})/{q}^{2}]}^{-1/2}.$$

**Figure 3:**
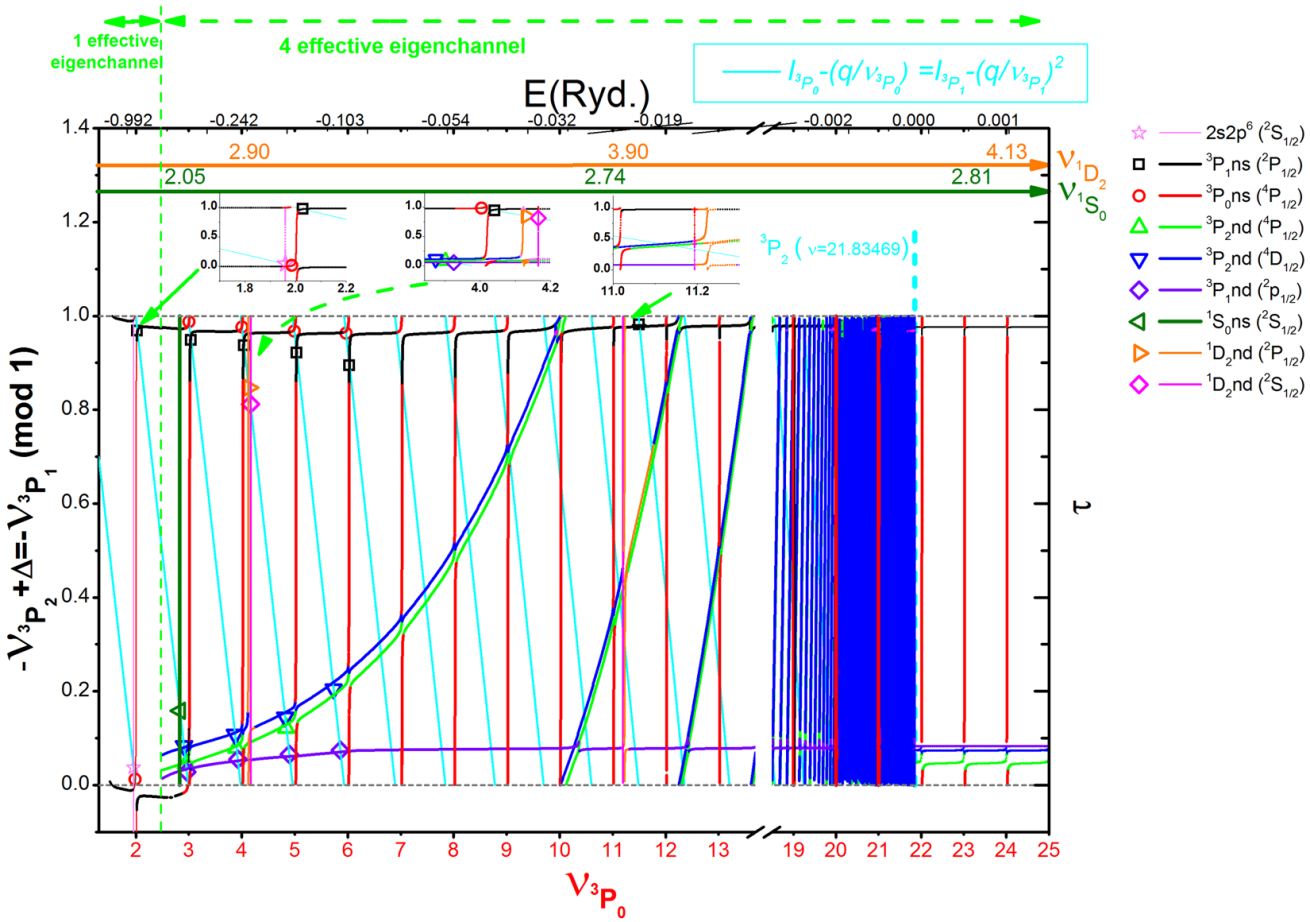
Projected high dimensional quantum-defect graph (symmetrized). Four color branch curves (black, blue, green, violet) are solutions of Eq. (5) under energy constrain Eq. (7). Intersection points between the solutions and light-blue line sections for Eq. (4b) should be all energy levels corresponding to the observed data. It’s convenient to assign various energy levels with the plot systematically. The locally isolated states which form Rydberg series converging to the other higher thresholds ^1^ *D*_2_ and ^1^ *S*_0_, can be clearly seen with the auxiliary abscissa ${\nu }_{{}^{1}D_{2}}$ and ${\nu }_{{}^{1}S_{0}}$ on the top.

As shown in Fig. 3, because of the constrain, two blue and green branch curves converging to the threshold ${}^{3}P_{2}$ go up-tilted as energy increases, the other two black and violet branch curves associated with the threshold ${}^{3}P_{1}$ appear as “horizontal” and smoothly cross the ^3^ *P*_2_ threshold. All the available experimental data^38^ (${\nu }_{{}^{3}{P}_{0}}^{\exp .}$, ${\nu }_{{}^{3}{P}_{1}}^{\exp .}$) marked as colored symbols lie on the crossing points between the four branch curves and the cyan line sections (energy relation between ^3^ *P*_1_ and ^3^ *P*_0_). With the Fig. 3 regarded as a projected high dimensional quantum-defect graph (symmetrized), one can easily compare the theoretical calculation with spectroscopic energy levels (${\nu }_{{}^{3}{P}_{0}}^{\exp .}$, ${\nu }_{{}^{3}{P}_{1}}^{\exp }.$) to check/calibrate the calculated MQDT parameters with desired accuracies. The calculated energy levels are in good agreement with experimental spectroscopic data by adjusting the MQDT parameters only within a few percent as shown in Fig. 1. Furthermore, for this case with many overlapping resonances, the Rydberg levels associated with different thresholds can be systematically and properly assigned with the plot as shown in the legend. It’s interesting to note that our assignments for some states are different from the NIST data^38^, such as the ${}^{3}P6d({}^{4}P_{1/2},{}^{4}D_{1/2})$ and ^1^ *D*4*d*(^2^ *S*_1/2_) states in the NIST assignments, which deserve further experimental observations. In order to see the isolated states clearly, we also plot ${\nu }_{{}^{1}D_{2}}$ and ${\nu }_{{}^{1}S_{0}}$ as auxiliary abscissas, from which one can easily see that locally isolated states form Rydberg series converging to the thresholds ^1^ *D*_2_ and ^1^ *S*_0_. Higher isolated states of these Rydberg series should be observed in the autoionization continuum energy region and will be discussed later. It’s worth to note that, the choice of the adjacent *I*_*one*_ and *I*_*two*_ is according to physical requirements. If we choose *I*_*one*_ as the lowest threshold of the ion core, the additional constrain conditions are dummy. In this case, the projected high dimensional quantum-defect graph (symmetrized) method and the recursive projection method are equivalent. Therefore our proposed pro***j***ected ***h***igh dimensional qu***an***tum-defect ***g***raph (symmetri***z***ed), i.e., JHANGZ plot, can be regarded as a general method for the multi-thresholds problems.

### Application to Ne^+^ resonant photoionization processes and demonstration of the merits of the method

In the autoionization region, there are *N*_*c*_ number of eigenchannels with negtive orbital energy and *N*_*o*_ number of eigenchannels with positive orbital energy, which satisfy closed channel boundary condition and open channel boundary condition respectively. The asymptotic boundary conditions require^18, 25–33^,

8 $$\{\begin{array}{ll}\sum _{\alpha }{U}_{i\alpha }\,\sin \,\pi ({\nu }_{i}+{\mu }_{\alpha }){A}_{\alpha }^{\rho }=0 & {\rm{for}}\,{N}_{c}\,{\rm{closed}}\,i,\\ \sum _{\alpha }{U}_{i\alpha }\,\sin \,\pi (-{\tau }^{\rho }+{\mu }_{\alpha }){A}_{\alpha }^{\rho }=0 & {\rm{for}}\,{N}_{o}\,{\rm{opened}}\,i{\rm{.}}\end{array}$$

Here *τ* ^*ρ*^ is the effective eigenchannel quantum defects (i.e., collisional eigenchannel phase shifts), which correspond to the effective eigenchannel quantum defects in discrete energy region. The oscillator strength density *df*/*dE* can be obtained as,

9 $$\frac{df}{dE}=\sum _{\rho }^{{N}_{o}}\frac{{\rm{d}}{f}^{(\rho )}}{{\rm{d}}E}=\sum _{\rho }^{{N}_{o}}\frac{2(E-{E}_{0}){|{\sum }_{\alpha }{D}_{\alpha }{A}_{\alpha }^{\rho }|}^{2}}{{N}_{\rho }^{2}},$$

with the reduced dipole matrix elements ***D***_*α*_ = 〈*ψ*_*α*_| |***D***| |Ψ_*o*_〉, and the normalization factor ${N}_{\rho }=\sqrt{{\sum }_{i}^{{N}_{o}}{[{\sum }_{\alpha }{U}_{i\alpha }\cos \pi (-{\tau }_{\rho }+{\mu }_{\alpha }){A}_{\alpha }^{\rho }]}^{2}}$ ^18, 25–33^.

**Figure 4:**
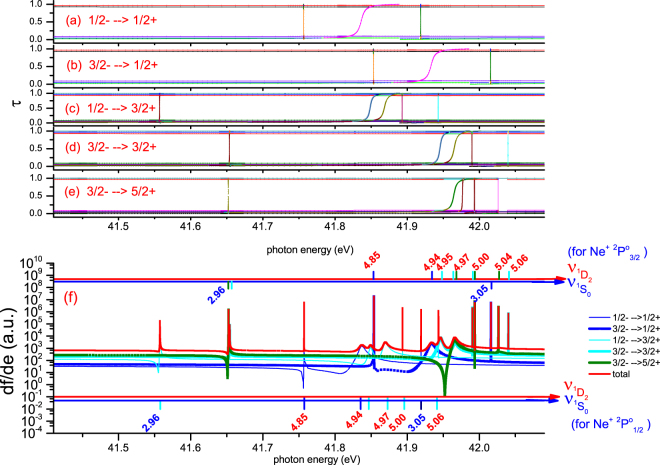
Effective eigenchannel quantum defects (collisional eigenchannel phase shifts) and the oscillator strength densities from different initial state to final channel symmetries. Because the abscissa is photon energy, the resonances from 1/2^−^ initial state is 0.096 eV lower than that from 3/2^−^ as shown in (**a**–**e**) respectively. (**f**) is the corresponding oscillator strength densities.

**Figure 5:**
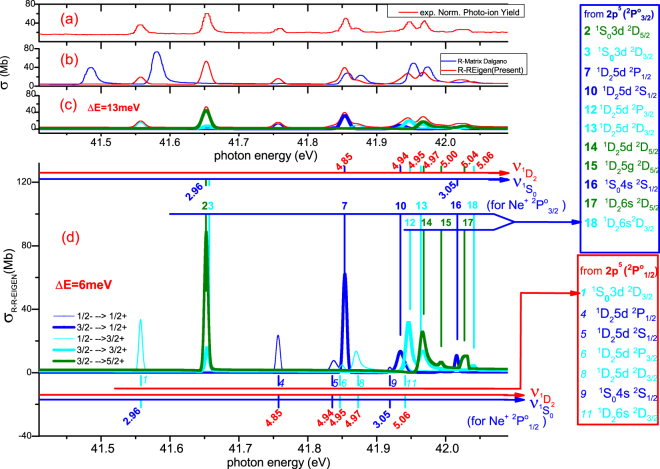
Photoionization cross section of Ne^+^. (**a**) Normalized experimental photo-ion Yields of Ne^+^ photoionization from the $\,2{p}^{5}\,{}^{2}P_{1/2,3/2}^{o}$; (**b**) Red: the R-R-Eigen calculation of total cross section (convoluted with effective energy resolution Δ*E* = 13 *meV*); Blue: the R-Matrix calculation^34^ (with Δ*E* = 11 *meV*). (**c**) Decomposition our calculation in (**b**). (**d**) Decomposition of total theoretical cross section with Δ*E* = 6 *meV* in order to show the resonances clearly. The auxiliary abscissas ${\nu }_{{}^{1}D_{2}}$ (red coordinate) and ${\nu }_{{}^{1}S_{0}}$ (blue coordinate) are plotted on the top and bottom of Fig. 5(d) to help identify the resonances from initial states $\,2{p}^{5}\,{}^{2}P_{3/2}^{o}$ and $\,2{p}^{5}\,{}^{2}P_{1/2}^{o}$ respectively. The assignments of all complex overlapping sharp resonances are summarized as the two legends shown on the right of the figure.

With the MQDT parameters (i.e., {*μ*_*α*_, *U*_*iα*_} as well as the dipole matrix elements *D*_*α*_) checked/calibrated with the precise experimental data in both discrete and continuum energy ranges, one can study various dynamic processes such as the electron-atomic ion collisions. We return to examine photoionization processes of Ne^+^ from both the $\,2{p}^{5}\,{}^{2}P_{1/2}^{o}$ and $\,2{p}^{5}\,{}^{2}P_{3/2}^{o}$ initial states. With the calculated MQDT parameters for all 1/2^+^, 3/2^+^, 5/2^+^ final channels, photoionization cross sections, i.e., the oscillator strength densities^21^ for all eigenchannels are calculated. As shown in Fig. 4(a–e) the effective eigenchannel quantum defects are calculated for all final channels respectively with the corresponding initial states. Note that because the abscissa is photon energy the resonances from 1/2^−^ initial state is 0.096 eV lower than that from 3/2^−^ for the same final channel. Figure 4(f) shows our calculated total oscillator strength density *df*/*dE* and *df* ^(*ρ*)^/*dE*. As can be seen, the origin of resonances can be easily identified from the resonant phase shifts in Fig. 4(a–e) with the help of calculated mixing coefficients ${A}_{\alpha }^{\rho }$ as well as ${{\nu }_{1}}_{{D}_{2}}$, ${{\nu }_{1}}_{{S}_{0}}$ plotted as auxiliary abscissa on the top and bottom of Fig. 4(f) for resonances from 1/2^−^ and 3/2^−^ initial states respectively. The assignments are shown in the right legends of Fig. 5 explicitly.

Figure 5(b) displays our calculated total cross section as the red line with effective energy resolution Δ*E* = 13 *meV*, by which the major experimental width and the minor radiative width are included along with the autoionization width having been taken into account adequately in our calculations. As a comparison, the R-Matrix calculation^34^ are plotted as blue line in Fig. 5(b) with Δ*E* = 11 *meV*. Our calculation agrees well with the normalized experimental photo-ion yields of Ne^+^ photoionizations shown in Fig. 5(a), which have been observed at the synchrotron radiation light source ALS^34^ with the line width Δ*E* = 11 *meV*. In Fig. 5(c) we decompose our R-R-eigen calculated total cross section shown in Fig. 5(b) into partial cross sections from both $\,2{p}^{5}\,{}^{2}P_{1/2}^{o}$ and $\,2{p}^{5}\,{}^{2}P_{3/2}^{o}$ initial states to three different final state symmetries as denoted in legends. In order to identify the sharp overlapping resonances systematically and more clearly, the partial cross sections are convoluted with a narrower effective energy resolution Δ*E* = 6 *meV* as shown in Fig. 5(d). Assignments of such overlapping sharp resonances are made from the calculated mixing coefficients of the eigenchannels *A*_*α*_, which are listed in the legends at the right hand side of Fig. 5. The auxiliary abscissas ${\nu }_{{}^{1}D_{2}}$ (red coordinate) and ${\nu }_{{}^{1}S_{0}}$ (blue coordinate) are also plotted on the top and bottom of Fig. 5(d) to help identify the resonances from both $\,2{p}^{5}\,{}^{2}P_{3/2}^{o}$ and $\,2{p}^{5}\,{}^{2}P_{1/2}^{o}$ initial states respectively.

## Conclusion

We would like to conclude by the following remarks. With the R-R-Eigen code, we calculated the short-range scattering matrices (i.e., the MQDT parameters {*μ*_*α*_, *U*_*iα*_}) and the corresponding eigenchannel dipole matrix elements *D*_*α*_ with good analytical properties in the whole energy regions for 1/2^+^, 3/2^+^ and 5/2^+^ symmetry block of Ne^+^ respectively. Note that one should choose an appropriate set of *n*_*p*_ physical channels to guarantee the analytical continuation property of scattering matrices as shown in Fig. 1. If one would choose an inappropriate set of *n*_1_ < *n*_*p*_ physical channels as the *n*_1_ effective physical channels, one then could calculate the *n*_1_ effective eigenchannels scattering matrices {*τ* ^*ρ*^, *T*_*i*_′_*ρ*_} with R-R-Eigen code as shown in Fig. 2(a). It would take greater efforts to scan all these resonant structures with a much finer energy grid. The calculated *τ* ^*ρ*^ are equivalent to effective eigen-quantum-defects obtained from MQDT procedure^23^ based on scattering matrices from full physical channel calculations. For the multi-thresholds problem shown in this work, we proposed a pro***j***ected ***h***igh dimensional qu***an***tum-defect ***g***raph (symmetri***z***ed), i.e JHANGZ plot, as an extension of Lu-Fano plot for multi-thresholds problems (more than two) to semi-analytically and graphically calculate all discrete levels without missing any one. As shown in Fig. 3, all discrete levels are calculated without missing any one. From the behavior of branch curves, one can assign the energy levels readily on equal footing. For example, the channels associated with ^3^ *P*_1_ behave as horizontal curves, and the channels associated with ^3^ *P*_2_ just go up-tilted as energy approaches the threshold ^3^ *P*_2_, channels associated with ^3^P_0_ behave as periodic vertical resonances at integer ${\nu }_{{}^{3}P_{0}}$, channels associated with higher thresholds ^1^ *D*_2_ and ^1^ *S*_0_ behave as locally isolated resonances. It’s also interesting to note that our assignments for some states are different from the NIST data^38^. For example, we suggest the ^3^ *P*6*d*(^4^ *P*_1/2_, ^4^ *D*_1/2_) and ^1^ *D*4*d*(^2^ *S*_1/2_) states in the NIST assignments should be assigned to ^3^ *P*_2_6*d*(^4^ *D*_1/2_), ^3^ *P*_1_6*d*(^2^ *P*_1/2_) and ^3^ *P*_1_ 13*s*(^4^ *P*_1/2_) respectively. Only with these assignments, the blue open triangle and violet diamond at ${\nu }_{{}^{3}p_{0}}\approx 6$ and black square at ${\nu }_{{}^{3}P_{0}}\approx 11.5$ will be in the same blue up-tilted violet and black horizontal curves respectively. We suggest further experimental verifications for these states. On the other hand, through the analytical property, the MQDT parameters in discrete/autoionization region can be readily compared with the spectroscopy data/autoionization spectra in the graph, by which the scattering matrices are checked/calibrated. Then autoionization profiles with all the sharp overlapping resonances can be calculated and assigned properly as shown in Fig. 5(b), in excellent agreement with the precise ALS experiment^34^ shown in Fig. 5(a). With the energy levels and autoionization profiles calculated, it should also be helpful to resonance ionization spectroscopy (RIS) experimental designs in choosing the appropriate intermediate state and laser wavelength^39^. We note that there are some theoretical studies on complex overlapping resonances using analytical formulations based on the phase-shifted MQDT parameters^40, 41^ and configuration interactions (CI) method^42^. Compared with these methods, the MQDT physical parameters are directly calculated in the R-R-eigen method and no fitting parameters are needed. Furthermore, the more general multi-threshold cases can be dealt with using the JHANGZ plot. Therefore, in the R-R-Eigen method, with the scattering matrices checked/calibrated against spectroscopy data in both discrete and continuum energy region, all important energy levels and related dynamic processes such as the electron-atomic ion collisions, photo-ionizations and autoionizations as well as its inverse processes (i.e., the dielectronic recombination) should be calculated with desired accuracies on equal footing. Hopefully, one can unravel the above mentioned dichotomy in nebular research: the abundances of metal ions “X” (such as Oxygen) determined by optical recombination lines(ORLs) are much higher than that determined by collisional excitation lines(CELs)^1^. In the precision physics stage, all important levels and related processes should be studied with adequate accuracy for the simulation and diagnosis of fusion plasmas (including inertial confinement and magnetic confinement fusions) in energy researches^43–45^. Therefore in the scenario, with some benchmark experiments, the R-R-Eigen method should be indispensable in the study of basic dynamic processes and ‘complete set’ of physical parameters, which are vitally important in astrophysics and inertial confinement research (Note that in inertial confinement research, one may need to properly consider the high density environmental effects^46, 47^).

### Data availability statement

The datasets generated during and/or analysed during the current study are available from the corresponding author on reasonable request.
